# Supplementary material for: Heat adaptation of phage T7 under an extended genetic code
Source: Virus Evol. 2021 Dec 1;7(2):veab100. doi: 10.1093/ve/veab100 (PMC8923235; doi:10.1093/ve/veab100)
Supplement: veab100_Supp [file veab100_supp.zip › Sequencing_Analysis.zip!/Sequencing_Analysis/A3/evidence/JC_52.html]

BRESEQ :: Evidence


| New junction evidence | | | | | | | | | | | |
| --- | --- | --- | --- | --- | --- | --- | --- | --- | --- | --- | --- |
|  | | seq id | position | reads (cov) | reads (cov) | score | skew | freq | annotation | gene | product |
| \* | ? | NC\_001604.1 | = 281 | 1741 (1.120) | 373 (0.220) +TAAAGACCAGACC | 41/508 | 22.1 | 16.7% | intergenic (–/–) | *–/–* | –/– |
|  | ? | NC\_001604.1 | 282 = | 1933 (1.110) |  |  |  |  | intergenic (–/–) | *–/–* | –/– |
| Rejected: Coverage evenness skew score above cutoff. | | | | | | | | | | | |
| Rejected: Frequency below/above cutoff threshold. | | | | | | | | | | | |

Only 100 of 377 total aligned reads displayed.  

`‑‑‑‑‑‑‑‑‑‑‑‑‑‑TCTCACAGTGTACGGACCTAAAGTTCCCCCATA‑GGGGGTACCTAAAGCCCAGCCAATCACCTAAAGTCAACCTTCGGTTGACCTTGAGGGTTCCCTAAGGGTTGGGGATGACCCTT‑GGGTTTGTCTTTGGGTGTTACCTTGAGTGTCTCTCTGTG‑T‑C‑CCTATCTGTTACAGT‑‑‑‑‑‑‑‑‑‑‑‑‑CTCCTAAAGTATCCTCCTAAAGTCACCTCCTAACGTCCATCCTAAAGCCAACACCTAAAGCCTACACCTAAAGACCCATCAAGTCAACGCCTATCTTAAAGTTTAAACA‑‑‑‑‑‑‑‑‑‑‑‑‑‑‑‑‑‑‑‑‑‑‑‑‑‑‑‑‑‑‑‑‑‑‑‑‑‑‑‑‑‑‑‑‑‑‑‑‑‑‑‑‑‑‑‑‑‑‑‑‑‑‑‑‑‑‑‑‑‑‑‑‑‑‑‑‑‑‑‑‑‑‑‑‑‑‑‑‑‑‑‑‑‑‑‑‑‑‑‑‑‑‑‑‑‑‑‑‑‑‑‑‑‑‑‑‑‑‑‑‑‑‑‑‑‑‑‑‑‑‑‑‑‑‑‑‑‑‑‑‑‑‑‑‑‑‑‑‑‑‑‑‑‑‑‑‑‑‑‑‑‑‑‑‑‑‑‑‑‑‑‑‑‑‑‑‑‑‑‑‑‑‑‑‑‑‑‑‑‑‑‑‑‑‑‑‑‑‑‑‑‑‑‑‑‑‑‑‑‑‑‑‑‑‑‑‑‑‑‑‑‑‑‑‑‑‑‑‑‑‑‑‑‑‑‑‑‑‑‑‑‑‑‑‑‑‑‑‑‑‑‑‑‑‑‑‑‑‑‑‑‑‑‑‑‑‑‑‑‑‑‑‑‑‑‑‑‑‑‑‑‑‑‑‑‑‑‑‑‑‑‑‑‑‑‑‑‑‑‑‑‑‑‑‑‑‑‑‑‑‑‑‑‑‑‑‑‑‑‑‑‑‑  >  NC_001604.1/1‑281`  
`‑‑‑‑‑‑‑‑‑‑‑‑‑‑‑‑‑‑‑‑‑‑‑‑‑‑‑‑‑‑‑‑‑‑‑‑‑‑‑‑‑‑‑‑‑‑‑‑‑‑‑‑‑‑‑‑‑‑‑‑‑‑‑‑‑‑‑‑‑‑‑‑‑‑‑‑‑‑‑‑‑‑‑‑‑‑‑‑‑‑‑‑‑‑‑‑‑‑‑‑‑‑‑‑‑‑‑‑‑‑‑‑‑‑‑‑‑‑‑‑‑‑‑‑‑‑‑‑‑‑‑‑‑‑‑‑‑‑‑‑‑‑‑‑‑‑‑‑‑‑‑‑‑‑‑‑‑‑‑‑‑‑‑‑‑‑‑‑‑‑‑‑‑‑‑‑‑‑‑‑‑‑‑‑‑‑‑‑‑‑‑‑‑‑‑‑‑‑‑‑‑‑‑‑‑‑‑‑‑‑‑‑‑‑‑‑‑‑‑‑‑‑‑‑‑‑‑‑‑‑‑‑‑‑‑‑‑‑‑‑‑‑‑‑‑‑‑‑‑‑‑‑‑‑‑‑‑‑‑‑‑‑‑‑‑‑‑‑‑‑‑‑‑‑‑‑‑‑‑‑‑‑‑‑‑‑‑‑‑‑‑‑‑‑‑‑‑‑‑‑‑‑‑‑‑‑‑‑‑‑‑‑‑‑‑‑‑‑‑‑‑‑‑‑‑‑‑‑‑‑‑‑‑‑‑‑‑‑‑TAAAGACCAGACCTAAAGACCAGACCTAAAGACACTACATAAAGACCAGACCTAAAGACGCCTTGTTGTTAGCCATAAAGTGATAACCTTTAATCATTGTCTTTATTAATACAACTCACTATAAGGAGAGACAACTTAAAGAGACTTAAAAGATTAATTTAAAATTTATCAAAAAGAGTATTGACTTAAAGTCTAACCTATAGGATACTTACAGCCATCGAGAGGGACACGGCGAATAGCCATCCCAATCGACACCGGGGTCAACCGGATAAGTAGACAGCCTGATAAGTCGCACGA  >  NC_001604.1/282‑578`  

                                                                                                                                                                                                                                                                                                                         `||||||||||||||||||||||||||`                                                                                                                                                                                                                                                                                                           
`ggagatgtgtataagagacagggggACGGACCTAAAGTTCCACCATA‑GGGGGTACCTAAAGCCCATCCAATCACCTAAAGTCAACCTTCGGTTGACCTTGAGGGTTCCATAAGGGTTGGGGATGACCCTT‑GGGTTTGTCTTTGGGTGTTACCTTGAGTGTCTCTCTGTG‑T‑C‑CCTATCTG‑TACAGT‑‑‑‑‑‑‑‑‑‑‑‑‑CTCCTAAAGTATCCTCCTAAAGTCACCTCCTAACGTCCATCCTAAAGCCAACACCTAAAGCCTACACCTAAAGACCCATCAAGTCAACGCCTATCTTAAAGTTTAAACA‑‑‑‑‑‑‑‑‑‑‑‑‑TAAAGAC                                                                                                                                                                                                                                                                                                                 <  1:26318/276‑1`  
 `gagaagtgtataagagacagtTGTACGGACCTAAAGTTCCCCCATA‑GGGGGTACCTAAATCCCAGCCAATCACCTAAAGTCAACCTTCGGTTGACCTTGAGGGTTCCCTAAGGGTTGGGGATGACCCTT‑GGGTTTGTCTTTGGGTGTTACCTTGAGTGTCTCTCTGTG‑T‑C‑CCTATCTGTAACAGT‑‑‑‑‑‑‑‑‑‑‑‑‑CTCCTAAAGTATCCTCCTAAAGTCACCTCCTAACGTCCATCCTAAAGACAACACCTAAAGCCTACACCTAAAGACCCATCAAGTCAACGCCTATCTTAAAGTTTAAACA‑‑‑‑‑‑‑‑‑‑‑‑‑TAAAGAC                                                                                                                                                                                                                                                                                                                 <  1:91116/280‑1`  
 `cggagatgtgtataagagacagGTACGGACCTAAAGTTCCCCCATA‑TGGGGTACCTAAAGCCCAGCCAATCACCTAAAGTCAACCTTCGGTTGACCTTGAGGGTTCCCTAAGGTTTGGGGATGACCCTT‑GGGTTTGTCTTTTGGTGTTACCTTGAGTGTCTCTCTGTG‑T‑C‑CCTATCTGTAACAGT‑‑‑‑‑‑‑‑‑‑‑‑‑CTCCTAAAGTATCCTCCTAAAGTCACCTCCTAACGTCCATCCTAAAGCCAACACCTAAAGCCTACACCTAAAGACCCATCAAGTCAACGCCTATCTTAAAGTTTAAACA‑‑‑‑‑‑‑‑‑‑‑‑‑TAAAGAC                                                                                                                                                                                                                                                                                                                 <  1:7549/279‑1`  
  `ggctcggagatgtgtataagagacaGGACCTAAAGTGCCCCCATA‑GGGGGTACCTAAAGCCCAGCCAATCACCTAAAGTCAACCTTCGGTTGACCTTGAGGGTTCCCTAAGGGTGGGGGATGACCCTT‑GGGTTTGTCTGTGGGTGTTACCTGGAGTGTCTCTCTGTG‑T‑C‑CCTATCTGTAACAGT‑‑‑‑‑‑‑‑‑‑‑‑‑CTCCTAAAGTATCCTCCTAAAGTCACCTCCTAACGTCCAGCCTAAAGCCAACACCTAAAGCCTACACCTAAAGACCCATCAAGTCAACGCCTATCTTAAAGTTTAAACA‑‑‑‑‑‑‑‑‑‑‑‑‑TAAAGACC                                                                                                                                                                                                                                                                                                                <  1:245825/276‑1`  
   `gatgtgtataagagacagGTGTACGGACCTAAAGTTCCCCCATA‑GGGGGTACCTAAAGCCCAGCAAATCACCTAAAGTCAACCTTCGGTTGACCTTGAGGGTTCCCTAAGGGTTGGGGATGACCCTT‑GGGTTTGTCTTTGGGTGTTACCTTGAGTGTCTCTCTGTG‑TCC‑CCTATCTGTTACAGT‑‑‑‑‑‑‑‑‑‑‑‑‑CTCCTAAAGTATCCTCCTAAAGTCACCTCCTAACGTCCATCCTAAAGCCAACACCTAAAGCCTACACCTAAAGACCCATCAAGTCAACGCCTATCTTAAAGTTTAAACA‑‑‑‑‑‑‑‑‑‑‑‑‑TAAAGACC                                                                                                                                                                                                                                                                                                                <  1:61029/283‑1`  
           `taagagacaggttgACGGACCTAAAGTTCCCCCATA‑GGGGGTACCTAAAGCCCAGCCAATCACCTAAAGTCAACCTTCGGTTGACCTTGAGGGTTCCCTAAGGGTTGGGGATGACCCTT‑GGGTTTGTCTTTGGGTGTTACCTTGAGTGTCTCTCTGTG‑T‑C‑CC‑ATCTGTTACAGT‑‑‑‑‑‑‑‑‑‑‑‑‑CTACTAAAGTATCCTCCTAAAGTCACCTCCTAACGTCCATCCTAAAGCCAACACCTAAAGCCTACACCTAAAGACCCATCAAGTCAACGCCTATCTTAAAGTTTAAACA‑‑‑‑‑‑‑‑‑‑‑‑‑TAAAGACCAGACCTAAAG                                                                                                                                                                                                                                                                                                      <  1:263749/287‑1`  
           `ggagcagcagGTGAACGGACCTAAAGTTCCCCCAAA‑GGGGGTACCTAAAGCCCAACCAATCACCTAAAGGCAACCTTCGGTTGACCTTGAGGGTTCCCTAAGGGTTGGGGATGACCCTT‑GGGTTTGTCTTTGGGTGTTACCATGAGTGTCTCTCTGTG‑T‑C‑CCTATCTG‑TACAGT‑‑‑‑‑‑‑‑‑‑‑‑‑CTCCTAAAGTATCCTCCTAAAGTCACCTCCTAACGTCCATCCTAAAGCCAACACCTAAAGCCTACACCTAAAGACCCATCAAGTCAACGCCTATCTTAAAGTTTAAACA‑‑‑‑‑‑‑‑‑‑‑‑‑TAAAGACCAGACCTAAAG                                                                                                                                                                                                                                                                                                      <  1:111373/291‑1`  
           `aaagggacagGTGTACGGACCTAAAGGTCCCCCATA‑GGGGGTACCTAAAGCCCGGCCAATCACCTAAAGTCAACCTTCGGTTGACCTTGAGGGTTCCCTAAGGGTTGTGGATGACCCTT‑GGGTTTGTCTTTGGGTGTTACCTTGAGTGTCTCTCTGTG‑T‑C‑CC‑ATCTGTTACAGT‑‑‑‑‑‑‑‑‑‑‑‑‑CTCCTAAAGTATCCTCCTAAAGTCACCTCCTAACGTCCATCCTAAAGCCAACACCTAAAGCCTACACCTAAAGACCCATCAAGTCAACGCCTATCTTAAAGTTTAAACA‑‑‑‑‑‑‑‑‑‑‑‑‑TAAAGACCAGACCTAAAG                                                                                                                                                                                                                                                                                                      <  1:176250/291‑1`  
            `aagtggtaaaaagaaaagCCTAAAATTCCCCCAAA‑GGGGGTAACTTAAGGCCAGCCAATCAACTAAAGGCAAACGTCGGTTGACCTTGAGGGTTGCCCAAGGGTTGGGGATGACCCTT‑GGGGTTGTCTTTGGGTGTTAACTTGAGGGTCTCCCTGTG‑G‑C‑CCTATCTGTAACAGT‑‑‑‑‑‑‑‑‑‑‑‑‑CTCCTAAAGTATCCTCCTAAAGTCACCTCCTAACGTCCATCCTAAAGCCAACACCTAAAGCCTACACCTAAAGACCCATCAAGTCAACGCCTATCTTAAAGTTTAAACA‑‑‑‑‑‑‑‑‑‑‑‑‑TAAAGACCAGACCTAAAG                                                                                                                                                                                                                                                                                                      <  1:102410/283‑1`  
            `aagagacagGTGTACGGACCTAAAGTTCCCCCATA‑GGGGTTACCTAAAGCCCAGCCAATCACCTAAAGTCAACCTTCGGTTGACCTTGAGGGTTCCCGAAGGGTTGGGGATGACCCTT‑GGGTTTGTCTTTGGGTGTTACCTTGAGTGTCTCTCTGTG‑T‑C‑CCTATCTGTAACAGT‑‑‑‑‑‑‑‑‑‑‑‑‑CTCCTAAAGTATCCTCCTAAAGTCACCTCCTAACGCCCATCCTAAAGCCAACACCTAAAGCCTACACCTAAAGACCCATCAAGTCAACGCCTATCTTAAAGTTTAAACA‑‑‑‑‑‑‑‑‑‑‑‑‑TAAAGACCAGACCTAAAG                                                                                                                                                                                                                                                                                                      <  1:56484/292‑1`  
             `taagagacagGTACGGACCTAAAGTTCCCCCATA‑GGGGGTACCAAAAGCCCAGCCAATCACCTACAGTCCACCTTCGGTTGACCTTGAGGGTTCCCTAAGGGTTGGGGATGACCCTT‑GGGTTTGTCTTTGGGTGTTACCTTGAGTGTCTCTCTGTG‑T‑C‑CCTATCTG‑TACAGT‑‑‑‑‑‑‑‑‑‑‑‑‑CTCCTAAAGTATCCTCCTAAAGTCACCTCCTAACGTCCATCCTAAAGCCAACACCTAAAGCCTACACCTAAAGACCCATCAAGTCAACGCCTATCTTAAAGTTTAAACA‑‑‑‑‑‑‑‑‑‑‑‑‑TAAAGACCAGACCTAAAGAC                                                                                                                                                                                                                                                                                                    <  1:300481/291‑1`  
             `gtgtataagagacaGGACCTAAAGTTCCCCCATA‑GGGGGTACCTAAAGCCCAGCCAATCACCTAAAGTCAACCTTCGGTTGACCTTGAGGGTTCCCTAAGGGTTGGGGATGACCCTT‑GGGTTTGTCTTTGGGTGTTACATTGAGTGTCTCTCTGTG‑TCC‑CCTATCTGTTACAGT‑‑‑‑‑‑‑‑‑‑‑‑‑CTCCTAAAGTATCCTCCTAAAGTCACCTCCTAACGTCAATCCTAAAGCCAACACCTAAAGCCTACACATAAAGACCCATCAAGTCAACGCCTATCTTAAAGTTTAAACA‑‑‑‑‑‑‑‑‑‑‑‑‑TAAAGACCAGACCTAAAG                                                                                                                                                                                                                                                                                                      <  1:277098/287‑1`  
             `agagacagggGTACGGACCTAAAGTTCCCCCATA‑GGGGGTACCTAAAGCCCAGCCAATCACCTAAAGTCAACCTTCGGTTGACCTTGAGGGTTCCCTAAGGGTTGGGGATGACCCTT‑GGGTTTGTCTTTGGGTGTTACCTTGAGTGTCTCTCTGTG‑T‑C‑CC‑ATCTGTTACAGT‑‑‑‑‑‑‑‑‑‑‑‑‑CTCCTAAAGTATCCTCCTAAAGTCACCTCCTAACGTCCATCCTAAAGCCAACACCTAAAGCCTACACCTAAAGACCCATCAAGTCAACGCCTATCTTAAAGTTTAAACA‑‑‑‑‑‑‑‑‑‑‑‑‑TAAAGACCAGACCTAAAGAC                                                                                                                                                                                                                                                                                                    <  1:295386/291‑1`  
              `gagacagGTGTACGGACCTAAAGTTCCCCCATA‑GGGGGTACCTAAAGCCCAGCCAATCACCTAAAGTCAACCTTCGGTTGACCTAGAGGGTTCCCTAAGGGTTGGTGATGACCCTT‑GGGTTTGTCTTTGGGTGTTACCTTGAGTGTCTCTCTGTG‑T‑C‑CCTATCTGTAACAGT‑‑‑‑‑‑‑‑‑‑‑‑‑CTCCTAAAGTATCCTCCTAAAGTCACCTCCTAACGTCCATCCTAAAGCCAACACCTAAAGCCTACACCTAAAGACCCATCAAGTCAACGCCTATCTTAAAGTTTAAACA‑‑‑‑‑‑‑‑‑‑‑‑‑TAAAGACCAGACCTAAAGAC                                                                                                                                                                                                                                                                                                    <  1:179242/294‑1`  
              `aagatacagGTACGTACCGAAAGTTCCCCCATA‑GGGGGTACCTAAAGCACAGCCAATCAACTCAAGTCAACCTTCGGTTGACCTTGAGGGTTCCCTAAGGGTTGGAGATGACCCTTAGGGTTTGTCTTTGGGTGTTACCTTGAGTGTCTCTCTGTG‑TCC‑CCTATCTGTTACAGT‑‑‑‑‑‑‑‑‑‑‑‑‑CTCCTAAAGTATCCTCCTAAAGTCACCCCCTAACGTCCATCCTAAAGCCAACACCTAAAGCCTACACCTAAAGACCCATCAAGTCAACGCCTATCTTAAAGTTTAAACA‑‑‑‑‑‑‑‑‑‑‑‑‑TAAAGACCAGACCTAAAG                                                                                                                                                                                                                                                                                                      <  1:240464/292‑1`  
              `aagagacaggtcCGGACCTAAAGTTCCCACATA‑GGGGGTACCTAAAGCCCAGCCAATCACCTAAAGTCAACCTTCGGTTGACCTTGAGGGTTCCCTAAGGGTTGGGGATGACCCTT‑GGGTTTGTCTTTGGGTGTTACCTTGAGTGTCTCTCTGTG‑T‑C‑CCTATCTGTAACAGT‑‑‑‑‑‑‑‑‑‑‑‑‑CTCCTAAAGTATCCTCCTAAAGTAACCTCCTAACGTCCATCCTAAAGCCAACACCTAAAGCCTACACCTAAAGACCCATCAAGTCAACGCCTATCTTAAAGTTTAAACA‑‑‑‑‑‑‑‑‑‑‑‑‑TAAAGACCAGACCTAAAGAC                                                                                                                                                                                                                                                                                                    <  1:271275/289‑1`  
               `gtataagagacaGGACCTAAAGGTCCCCCATA‑GGGGGTACCTAAAGCCCAGCCAAGCACCTAAAGTCAACCTTCGGTTGACCTTGAGGGTTCCCTAAGGGTTGGGGATGACCCTT‑GGGTTTGTCTTTGGGTGTTACCTTGAGTGTCTCTCTGTG‑TCC‑CCTATCTGTTACAGT‑‑‑‑‑‑‑‑‑‑‑‑‑CTCCTAAAGTATCCTCCTAAAGTCACCTCCTAACGTCCATCCTAAAGCCAACACCTAAAGCCTACACCTAAAGACCCATCAAGTCAACGCCTATCTTAAAGTTTAAACA‑‑‑‑‑‑‑‑‑‑‑‑‑TAAAGACCAGACCTAAAGAC                                                                                                                                                                                                                                                                                                    <  1:252383/289‑1`  
               `ctcggagatgtgtataagagacagCCCCCATA‑GGGGGTACCTAAAGCCCAGCCAATCACCTAAAGTCAACCTTCGGTTGACCTTGAGGGTTCCCTAAGGGTTGGGGATGACCCTT‑GGGTTTGTCTTTGGGTGTTACCTTGAGTGTCTCTCTGTG‑TCC‑CCTATCTGTTACAGT‑‑‑‑‑‑‑‑‑‑‑‑‑CTCCTAAAGTATCCTCCTAAAGTCACCTCCTAACGTCCATCCTAAAGCCAACACCTAAAGCCTACACCTAAAGACCCATCAAGTCAACGCCTATCTTAAAGTTTAAACA‑‑‑‑‑‑‑‑‑‑‑‑‑TAAAGACCAGACCTAAAGAC                                                                                                                                                                                                                                                                                                    <  1:166894/277‑1`  
               `agacagGTGTACGGACCTAAAGTTCCCCCATA‑GGGGGTACCTAAAGCCCAGCCAATCACCTAAAGTCAACCTTCGGTTGACCTTGAGGGTTCCCTAAGGGTTGGGGATGACCCTT‑GGGTTTGTCTTTGGGTGTTACCTTGAGTGTCTCTCTGTG‑T‑C‑CCTATCTGTAACAGT‑‑‑‑‑‑‑‑‑‑‑‑‑CTCCTAAAGTATCCTCCTAAAGTCACCTCCTAACGTCCATCCTAAAGCCAACACCTAAAGCCTACACCTAAAGACCCATCAAGTCAACGCCTATCTTAAAGTTTAAACA‑‑‑‑‑‑‑‑‑‑‑‑‑TAAAGACCAGACCTAAAGACC                                                                                                                                                                                                                                                                                                   <  1:257597/295‑1`  
                `gagacagGTACGGAACTAAAGTTCACCCAGA‑GGGGGTACCTAAAGCCCAGCCAATCACCTAAAGTCAACCTTCGGTTGACCTTGAGGGTTCCCTAAGGGTTGGGGATGACCCTT‑GGGTTTGTCTTTGGGTGTTACCTTGAGTGTCTCTCTGTG‑TCC‑CCTATCTGTTACAGT‑‑‑‑‑‑‑‑‑‑‑‑‑CTCCTAAAGTATCCTCCTAAAGTCACCTCCTAACGTCCATCCTAAATCCAACACCTAAAGCCTACACCTAAAGACCCATCAAGTCAACGCCTATCTTAAAGTTTAAACA‑‑‑‑‑‑‑‑‑‑‑‑‑TAAAGACCAGACCTAAAGACC                                                                                                                                                                                                                                                                                                   <  1:246659/294‑1`  
                    `gGTGTACTGACCTAAAGTTCACCCATA‑GGGGGTACCTAAAGCCCAGCCAATCACCTAAAGTCAACCTTCGGTTGACCTTGAGTGTTCCCTAAGGGTTGGGGATGACCCTT‑GGGTTTGTCTTTGGGTGTTACCTTGAGTGTCTCTCTGTG‑T‑C‑CCTATCTGTAACAGT‑‑‑‑‑‑‑‑‑‑‑‑‑CTCCTAAAGTATCCTCCTAAAGTCACCTCCTAACGTCCATCCTAAAGCCAACACCTAAAGCCTACACCTAAAGACCCATCAAGTCAACGCCTATCTTAAAGTTTAAACA‑‑‑‑‑‑‑‑‑‑‑‑‑TAAAGACCAGACCTAAAGACCAGACC                                                                                                                                                                                                                                                                                              <  1:110227/300‑1`  
                    `gGTGTACGGACCTAAAGTTCACCCATA‑GGGGGTACATAAAGCCCAGCCAATCACCTAAAGTCAACCTTCGGTTGACCTTGAGGGTTCCCTAAGGGTTGGGGATAACCCTT‑GGGTTTGTCTTTGGGTGTTACCTTGAGTGTCTCTCTGTG‑T‑C‑CCTATCTGTAACAGT‑‑‑‑‑‑‑‑‑‑‑‑‑CTCCTAAAGTATCCTCCTAAACTCACCTCCTAACGTCCATCCTAAAGCCAACACCTAAAGCCTACACCTAAAGACCCATCAAGTCAACGCCTATCTCAAAGTTTAAACA‑‑‑‑‑‑‑‑‑‑‑‑‑TAAAGACCAGACCTAAAGACCAGACC                                                                                                                                                                                                                                                                                              <  1:107887/300‑1`  
                     `gtgtaccacccacAAGTTCCCCCATA‑GGGGGTACCTAAAGCCCAGCCAATCACCTAAAGTCAACCTTCGGTTGACCTTGAGGGTTCCCTAAGGGTTGGGGATGACCCTT‑GGGTTTGTCTTTGGGTGTTACCTTGAGTGTCTCTCTGTG‑T‑C‑CCTATCTGTAACAGT‑‑‑‑‑‑‑‑‑‑‑‑‑CTCCTAAAGTATCCTCCTAAAGTCACCTCCTAACGTCCATCCTAAAGCCAACACCTAAAGCCTACACCTAAAGACCCATCAAGTCAACGCCTATCTTAAAGTTTAAACA‑‑‑‑‑‑‑‑‑‑‑‑‑TAAAGACCAGACCctgtctcttataca                                                                                                                                                                                                                                                                                             >  1:322330/14‑287`  
                     `GTGTATGGACCTAAAGTTCCCCCATA‑GGGGGTACCTAAAGCCCAGCCAATAACCTAAAGTCAAACTTCGGTTGACCTTGAGGGTTCCCTAAGGGTGGGGGATGACCCTT‑GGGTTTGTCTTTGGGTGTTACCTTGAGTGTCTCTCTGTG‑T‑C‑CCTATCTGTAACAGT‑‑‑‑‑‑‑‑‑‑‑‑‑CTCCTAAAGTATCCTCCTAAAGTCACCTCCTAACGTCCATCCTAAAGCCAACACCTAAAGCCTACACCTAAAGACCCCTCAAGTCAACGCCTATCTTAAAGTTTAACCA‑‑‑‑‑‑‑‑‑‑‑‑‑TAAAAACCAGACCTAACGCCCAGACCT                                                                                                                                                                                                                                                                                             >  1:43706/1‑301`  
                     `GTGTACTGACCTAAAGTTCCCCCATA‑GGGGGTACATAAAGCCCAGCCAATCACCTAAAGTCAACCTTCGGTTGACCTTGAGGGTTTCCTAAGGGTTGGGGATGACCCTT‑GGGTTTGTCTTTGGGTGTTACCTTGAGTGTCTCTCTGTG‑T‑C‑CCTATCTGTTACAGT‑‑‑‑‑‑‑‑‑‑‑‑‑CTCCTAAAGTATCCTCCTAAAGTAACCTCCTAACGTCCATCCTAAAGCCAACACCTAAAGCCTACACATAAAGACCCATCAAGTCAACGCCTATCTTAAAGTTTAAACA‑‑‑‑‑‑‑‑‑‑‑‑‑TAAAGACCAGACCTAAAGACCAGACCT                                                                                                                                                                                                                                                                                             >  1:547/1‑301`  
                     `GTGTACGGACCTAAAGTTCCCCCCTA‑GGGGGGACCTAAAGCCCAGCCAATCACCTAAAGTCAACCTTCGGTTGACCTTGAGGGTTCCCTAAGGGTTGGGGATGACCCTT‑GGGTTTGTATTTGGGTGTTACCTTGAGTGTCTCTCTGTG‑T‑C‑CCTAGCTGTAACAGT‑‑‑‑‑‑‑‑‑‑‑‑‑CTCCTAACGTATCATCCTAAAGTCACCTCCTAACGTCCATCCTAAAGCCAAAACCTAAAGCCTACACCTAAAGACCCATCAAGTCAACGCCTATCTTAAAGTTACAACA‑‑‑‑‑‑‑‑‑‑‑‑‑TAAAGACCAGACCTAAAGAACAaaact                                                                                                                                                                                                                                                                                             >  1:146477/1‑296`  
                     `GTGTACGGACCTAAAGTTCCCCCATA‑GGGGGTACCTAAAGTCCAGCCAATCACCTAAAGTCAACCTTCGTTTGACCTTGAGGGTTCCCTAAGGGTTGGGGATGACCCTT‑GGGTTTGTCTTTGGGTGTTAACTTGAGTGTTTTTCTGCG‑T‑CTCCTATATGTTACAGT‑‑‑‑‑‑‑‑‑‑‑‑‑CTCCTAAAGTGTCCTCCTAAAGTCACCTCCTAACGTCCATCCTAAAGCCAACACCTAAAGCCTACACCTCAAGACTCATCAAGTCAAAGCCTATCTTAAAGTTTAACCA‑‑‑‑‑‑‑‑‑‑‑‑‑TAACAACCAGACCTAAAGACTAGAac                                                                                                                                                                                                                                                                                              >  1:342490/1‑299`  
                     `GTGTACGGACCTAAAGTTCCCCCATA‑GGGGGTACCTAAAGCCCAGCCAATCACCTAAAGTCAACCTTCGGTTGACCTTGAGGGTTCCCTAAGGGTTGGGGATGACCCTT‑TGGTTTGTCTTTGGGTGTTACCTTGAGTGTCTCTCTGTG‑TCC‑CCTATCTGTTACAGT‑‑‑‑‑‑‑‑‑‑‑‑‑CTCCTAAAGTATCCTCCTAAAGTCACCTCCTAACGTCCATCCTAAAGCCAACACCTAAAGCCTACACCTAAAGACCCATCAAGTCAACGCCTATCTTAAAGTTTAAACA‑‑‑‑‑‑‑‑‑‑‑‑‑TAAAGACCAGACCTAAAGACCtgtct                                                                                                                                                                                                                                                                                              >  1:286719/1‑296`  
                     `GTGTACGGACCTAAAGTTCCCCCATA‑GGGGGTACCTAAAGCCCAGCCAATCACCTAAAGTCAACCTTCGGTTGACCTTGAGGGTTCCCTAAGGGTTGGGGATGACCCTT‑GGGTTTGTCTTTGGTTGTTACCTTGAGTGTCTCTCTGTG‑T‑C‑CCTATCTGTAACAGT‑‑‑‑‑‑‑‑‑‑‑‑‑CTCCTAAAGTATCCTCATAAAGTCACCTCCTAACGTCCATCCTAAAGCCAACACCTAAAGCCTACACCTAAAGACCCATCAAGTCAACGCCTATCTTAAAGTTTAAACA‑‑‑‑‑‑‑‑‑‑‑‑‑TAAAGACCtgtctcttatacacatctc                                                                                                                                                                                                                                                                                             >  1:231532/1‑282`  
                     `GTGTACGGACCTAAAGTTCCCCCATA‑GGGGGTACCTAAAGCCCAGCCAATCACCTAAAGTCAACCTTCGGTTGACCTTGAGGGTTCCCTAAGGGTTGGGGATGACCCTT‑GGGTTTGTCTTTGGGTGTTACCTTGAGTGTCTCTCTGTGCC‑C‑CCTATATGTTACAGT‑‑‑‑‑‑‑‑‑‑‑‑‑CTCCTAAAGTATCCTCCTAAAGTCACCTCCTAACGTCCATCCTAAAGCCAACACCTAAAGCCTACACCTAAAGACCCATCAAGTCAACGCCTATCTTAAAGTTTAAACA‑‑‑‑‑‑‑‑‑‑‑‑‑TAAAGACCctgtctcttatacccatc                                                                                                                                                                                                                                                                                              >  1:297538/1‑283`  
                     `GTGTACGGACCTAAAGTTCCCCCATA‑GGGGGTACCTAAAGCCCAGCCAATCACCTAAAGTCAACCTTCGGTTGACCTTGAGGGTTCCCTAAGGGTTGGGGATGACCCTT‑GGGTTTGTCTTTGGGTGTTACCTTGAGTGTCTCTCTGTG‑TCC‑CCTATCTGTTACAGT‑‑‑‑‑‑‑‑‑‑‑‑‑CTCCTAAAGTATCCTCCTAAAGTCACCTCCTAACGTCCATCCTAAAGCCAACACCTAAAGCCTACACCTAAAGACCCATCAATTCAACGCCTACCTTAAAGTTTAAACA‑‑‑‑‑‑‑‑‑‑‑‑‑TAAAGACAAGACCTAAAGACaaaccc                                                                                                                                                                                                                                                                                              >  1:1867/1‑295`  
                     `GTGTACGGACCTAAAGTTCCCCCATA‑GGGGGTACCTAAAGCCCAGCCAATCACCTAAAGTCAACCTTCGGTTGACCTTGAGGGTTCCCTAAGGGTTGGGGATGACCCTT‑GGGTTTGTCTTTGGGTGTTACCTTGAGTGTCTCTCTGTG‑TCC‑CCTATCTGTTACAGT‑‑‑‑‑‑‑‑‑‑‑‑‑CTCCTAAAGTATCCTCCTAAAGTCACCTCCTAACGTCCATCCTAAAGCCAACACCTAAAGCCTACACCTAAAGACCCATCAAGTCAACGCCTATCTTAAAGTTTAAACA‑‑‑‑‑‑‑‑‑‑‑‑‑TAAAGACCAGACCctgtctcttatac                                                                                                                                                                                                                                                                                              >  1:11937/1‑288`  
                     `GTGTACGGACCTAAAGTTCCCCCATA‑GGGGGTACCTAAAGCCCAGCCAATCACCTAAAGTCAACCTTCGGTTGACCTTGAGGGTTCCCTAAGGGTTGGGGATGACCCTT‑GGGTTTGTCTTTGGGTGTTACCTTGAGTGTCTCTCTGTG‑TCC‑CCTATCTGTTACAGT‑‑‑‑‑‑‑‑‑‑‑‑‑CTCCTAAAGTATCCTCCTAAAGTCACCTCCTAACGTCCATCCTAAAGCCAACACCTAAAGCCTACACCTAAAGACCCATCAAGTCAACGCCTATCTTAAAGTTTAAACA‑‑‑‑‑‑‑‑‑‑‑‑‑TAAAGACCAGACCTAAAGACCAGACC                                                                                                                                                                                                                                                                                              <  1:151304/301‑1`  
                     `GTGTACGGACCTAAAGTTCCCCCATA‑GGGGGTACCTAAAGCCCAGCCAATCACCTAAAGTCAACCTTCGGTTGACCTTGAGGGTTCCCTAAGGGTTGGGGATGACCCTT‑GGGTTTGTCTTTGGGTGTTACCTTGAGTGTCTCTCTGTG‑T‑C‑CCTATCTGTTACAGT‑‑‑‑‑‑‑‑‑‑‑‑‑CTCCTAAAGTATCCTCCTAAAGTCACCTCCTAACGTCCATCCTAAAGCCAACACCTAAAGCCTACTCCTAAAGACCCATCAAGTCAACGCCTATCTTAAAGTTTAAACA‑‑‑‑‑‑‑‑‑‑‑‑‑TAAAGACCAGACCTAAAGctgtctctt                                                                                                                                                                                                                                                                                             >  1:170978/1‑292`  
                     `GTGTACGGACCTAAAGTTCCCCCATA‑GGGGGTACCTAAAGCCCAGCCAATCACCTAAAGTCAACCTTCGGTTGACCTTGAGGGTTCCCTAAGGGTTGGGGATGACCCTT‑GGGTTTGTCTTTGGGTGTTACCTTGAGTGTCTCTCTGTG‑T‑C‑CCTATCTGTAACAGT‑‑‑‑‑‑‑‑‑‑‑‑‑CTCCTAAAGTATCCTCCTAAAGTCACCTCCTAACGTCCATCCTAAAGCCAACACCTAAAGCCTACACCTAAAGACCCATCAAGTCCACGCCTATCTTAAAGTTTAAACA‑‑‑‑‑‑‑‑‑‑‑‑‑TAAAGACCctgtctcttatacacatct                                                                                                                                                                                                                                                                                             >  1:63006/1‑282`  
                     `GTGTACGGACCTAAAGTTCCCCCATA‑GGGGGTACCTAAAGCCCAGCCAATCACCTAAAGTCAACCTTCGGTTGACCTTGAGGGTTCCCTAAGGGTTGGGGATGACCCTT‑GGGTTTGTCTTTGGGTGTTACCTTGAGTGTCTCTCTGTG‑T‑C‑CCTATCTGTAACAGT‑‑‑‑‑‑‑‑‑‑‑‑‑CTCCTAAAGTATCCTCCTAAAGTCACCTCCTAACGTCCATCCTAAAGCCAACACCTAAAGCCTACACCTAAAGACCCATCAAGTCAACGCCTATCTTAAAGTTTAAACA‑‑‑‑‑‑‑‑‑‑‑‑‑TAAAGACCAGACCTAAAGACCAGACCT                                                                                                                                                                                                                                                                                             >  1:117416/1‑301`  
                     `GTGTACGGACCTAAAGTTCCCCCATA‑GGGGGTACCTAAAGCCCAGCCAATCACCTAAAGTCAACCTTCGGTTGACCTTGAGGGTTCCCTAAGGGTTGGGGATGACCCTT‑GGGTTTGTCTTTGGGTGTTACCTTGAGTGTCTCTCTGTG‑T‑C‑CCTATCTGTAACAGT‑‑‑‑‑‑‑‑‑‑‑‑‑CTCCTAAAGTATCCTCCTAAAGTCACCTCCTAACGTCCATCCTAAAGCCAACACCTAAAGCCTACACCTAAAGACCCATCAAGTCAACGCCTATCTTAAAGTTTAAACA‑‑‑‑‑‑‑‑‑‑‑‑‑TAAAGACCAGACCTAAAGACCAGACCT                                                                                                                                                                                                                                                                                             >  1:104090/1‑301`  
                     `GTGTACGGACCTAAAGTTCCCCCATA‑GGGGGTACCTAAAGCCCAGCCAATCACCTAAAGTCAACCTTCGGTTGACCTTGAGGGTTCCCTAAGGGTTGGGGATGACCCTT‑GGGTTTGTCTTTGGGTGTTACCTTGAGTGTCTCTCTGTG‑T‑C‑CCTATCTGTAACAGT‑‑‑‑‑‑‑‑‑‑‑‑‑CTCCTAAAGTATCCTCCTAAAGTCACCTCCTAACGTCCATCCTAAAGCCAACACC‑AAAGCCTACACCTAAAGACCCATCAAGTCAACGCCTATCTTAAAGTTTAAACA‑‑‑‑‑‑‑‑‑‑‑‑‑TAAAGACCAGACCTAAAGACCAGACCTA                                                                                                                                                                                                                                                                                            >  1:204219/1‑301`  
                     `GTGTACGGACCTAAAGTTCCCCCATA‑GGGGGTACCTAAAGCCCAGCCAATCACCTAAAGTCAACCTTCGGTTGACCTTGAGGGTTCCCTAAGGGTTGGGGATGACCCTT‑GGGTTTGTCTTTGGGTGTTACCTTGAGTGTCTCTCTGTG‑T‑C‑CCTATCTG‑TACAGT‑‑‑‑‑‑‑‑‑‑‑‑‑CTCCTAAAGTATCCTCCTAATGTCACCTCCTAACGTCCATCCTAAAGCCAACACCTAAAGCCTACACCTAAAGACCCATCAAGTCAACGCCTATCTTAACGTTTAAACA‑‑‑‑‑‑‑‑‑‑‑‑‑TAAAGACCAGACCTAAAGACCctgtctc                                                                                                                                                                                                                                                                                            >  1:100958/1‑294`  
                     `GTGTACGGACCTAAAGTTCCCCCATA‑GGGGGTACCTAAAGCCCAGCCAATCACCTAAAGTCAACCTTCGGTTGACCTTGAGGGTTCCCTAAGGGTTGGGGATGACCCTT‑GGGTTTGTCTTTGGGTGTTACCTTGAGTGTCTCTCTGTG‑T‑C‑CC‑ATCTGTTACAGT‑‑‑‑‑‑‑‑‑‑‑‑‑CTCCTAAAGTATCCTCCTAAAGTCACCTCCTAACGTCCATCCTAAAGCCAACACCTAAAGCCTACACCTAAAGACCCATCAAGTCAACGCCTATCTTCAAGTTTAAACA‑‑‑‑‑‑‑‑‑‑‑‑‑TAAAGACCAGACCTAAAGACAAGACCTA                                                                                                                                                                                                                                                                                            >  1:335133/1‑301`  
                     `GTGTACGGACCTAAAGTTCCCCCATA‑GGGGGTACCTAAAGCCCAGCCAATCACCTAAAGTCAACCTTCGGTTGACCTTGAGGGTTCCCTAAGGGTTGGGGATGACCCTT‑GGGTTTGTCTTTGGGTGTTACCTTGAGTGTCTCTCTGTG‑T‑C‑CC‑ATCTGTTACAGT‑‑‑‑‑‑‑‑‑‑‑‑‑CTCCTAAAGTATCCTCCTAAAGTCACCTCCTAACGTCCATCCTAAAGCCAACACCTAAAGCCTACACCTAAAGACCCATCAAGTCAACGCCTATCTTAAAGTTTAAACA‑‑‑‑‑‑‑‑‑‑‑‑‑TAAAGACCAGACCTAAAGACCAGACCTA                                                                                                                                                                                                                                                                                            >  1:85659/1‑301`  
                     `GTGTACGGACCTAAAGTTCCCCCATA‑GGGGGTACCTAAAGCCCAGCCAATCACCTAAAGTCAACCTTCGGTTGACCTTGAGGGTTCCCTAAGGGTTGGGGATGACCCTT‑GGGTTTGTCTTTGGGTGTTACCTTGAGTGTCTCTCTGTC‑T‑C‑CCTATCTGTAACAGT‑‑‑‑‑‑‑‑‑‑‑‑‑CTCCTAAAGTATCCTCCTAAAGTCACCTCCTAACGTCCATCCTAAAGCCAACACCTAAAGCCTACACCTAAAGACCCATCAAGTCAACGCCTATCTTAAAGTTTAAACA‑‑‑‑‑‑‑‑‑‑‑‑‑TAAAGACCAGACCTAAAGACCAGACCT                                                                                                                                                                                                                                                                                             >  1:87625/1‑301`  
                     `GTGTACGGACCTAAAGTTCCCCCATA‑GGGGGTACCTAAAGCCCAGCCAATCACCTAAAGTCAACCTTCGGTTGACCTTGAGGGTTCCCTAAGGGTTGGGGATGACCCTT‑AGGTTTGTCTTTGGGTGTTACCTTGAGTGTCTCTCTGTG‑T‑C‑CCTATCTGTAACAGT‑‑‑‑‑‑‑‑‑‑‑‑‑CTCCTAAAGTATCCTCCTAAAGTCACCTCCTAACGTCCATCCTAAAGCCAACACCTAAAGCCTACACCTAAAGACCCATCAAGTCAACGCCTATCTTAAAGTTTAAACA‑‑‑‑‑‑‑‑‑‑‑‑‑TAAAGACCAGACCTAAAGctgtctctt                                                                                                                                                                                                                                                                                             >  1:13797/1‑292`  
                     `GTGTACGGACCTAAAGTTCCCCCATA‑GGGGGTACCTAAAGCCCAGCCAATCACCTAAAGTCAACCTTCGGTTGACCTTGAAGGTTCCCTAAGGGTTGGGGATGACCCTT‑GGGTTTGTCTTTGGGTGTTACCTTGAGTGTCTCTCTGTG‑TCC‑CCTATCTGTTACAGT‑‑‑‑‑‑‑‑‑‑‑‑‑CTCCTAAAGTATCCTCCTAAAGTCACCTCCTAACGTCCATCCTAAAGCCAACACCTAAAGCCTACACCTAAAGACCCATCAAGTCAACGCCTATCTTAAAGTTTAAACA‑‑‑‑‑‑‑‑‑‑‑‑‑TAAAGACCtgtctcttatacacatct                                                                                                                                                                                                                                                                                              >  1:265654/1‑283`  
                     `GTGTACGGACCTAAAGTTCCCCCATA‑GGGGGTACCTAAAGCCCAGCCAATCACCTAAAGACAACCTTCGGTTGACCTTGAGGGTTCCATAAGGGTTGGGGATGACCCTT‑GGGTTTGTCTTTGGGTGTTACCTTGAGTGTCTCTCTGTG‑T‑C‑CC‑ATCTGTTACAGT‑‑‑‑‑‑‑‑‑‑‑‑‑ATCCTAAAGTATCATCCTAAAGTCACCTCATAACATCAATCCTAAAGCAAACACCTAAAGCCTACCCCTAAAGACCCATCAAGTCAACGCCAATCTTAACGTTTAAACA‑‑‑‑‑‑‑‑‑‑‑‑‑TAAAGCTCAGCACTCAAGACCAGACTTA                                                                                                                                                                                                                                                                                            >  1:328247/1‑301`  
                     `GTGTACGGACCTAAAGTTCCCCCATA‑GGGGGTACCTAAAGCCAAGCCAATCACCTAAAGTCAACCTTCGGTTGACCTTGAGGGTTCCCTAAGGGTTGGGGATGACCCTT‑GGGTTTTTCTTTGGGTGTTACCTTGAGTGTCTCTCTGTG‑T‑C‑CC‑ATATGTTACAGT‑‑‑‑‑‑‑‑‑‑‑‑‑CTCCTAAAGTATCCTCCTAAAGTCACCTCCTAACGTCCATCCTAAAGCCAACACCTAAAGCCTACACCTAAAGACCCATCAAGTCAACGCCTATCTTAAAGTTTAAACA‑‑‑‑‑‑‑‑‑‑‑‑‑TAAAGACCAGACCTAAAGACCAGACCTA                                                                                                                                                                                                                                                                                            >  1:98451/1‑301`  
                     `GTGTACGGACCTAAAGTTCCCCCATA‑‑GGGGTACCTAAAGCCCAGCCAATCACCTAAAGTCAACCTTCGGTTGACCTTGAGGGTTCCCTAAGGGTTGGGGATGACCCTT‑GGGTTTGTCTTTGGGTGTTACCTTGAGTGTCTCTCTGTG‑T‑C‑CCTATCTG‑TACAGT‑‑‑‑‑‑‑‑‑‑‑‑‑CTCCTAAAGTATCCTCCTAAAGTCACCTCCTAACGTCCATCCTAAAGCCAACACCTAAAGCCTACACCTAAAGACCCATCAAGTCAACGCCTATCTTAAAGTTTAAACA‑‑‑‑‑‑‑‑‑‑‑‑‑TAAAGACCAGACCTAAAGACCAGACCTAA                                                                                                                                                                                                                                                                                           >  1:192406/1‑301`  
                     `GTGTACGGACCTAAAGTTCCCACATA‑GTGGGTACCTAAAGCCCAGCCAATCACCTAAACTCAACCTTCGGTTGACCTTGAGGGTTCCCTAAGGGTTGGGGATGACCCTT‑GGGTTTGTCTTTGGGTGTTACCTTGAGTGTCTCTCTGTG‑T‑C‑CCTATCTGTAACAGT‑‑‑‑‑‑‑‑‑‑‑‑‑CTCCTACAGTATCCTCCTAAAGCCACCTCCTAACGTCCATGCTAAAGCCAACACCTAAAGCCTACACCTAATGACCCATCCAGTCAACGCCTATCTTAACGTTTATACA‑‑‑‑‑‑‑‑‑‑‑‑‑TAAAGCCCAGACCTAAAGACCAGACCT                                                                                                                                                                                                                                                                                             >  1:159337/1‑301`  
                     `GTGTACGGACCTAAAGTTCCCACATA‑GGGGGTACCTAAAGCCCAGCCAATCACCTAAAGTCAACCTTCGGTCGACCTTGAGGGTTCCCTAAGGGTTGGGGATGACCCTT‑GGGTTTGTCTTTGGGTGTTACCTTGAGTGTCTCTCTGTG‑T‑C‑CCTATCTG‑TACAGT‑‑‑‑‑‑‑‑‑‑‑‑‑CTCCTAAAGTATCCTCCTAAAGTCCCCTCCTAACGTCCATCCTAAAGCCAACACCTAAAGCCTACACCTAAAGACCAATCAAGACAACGCCTATCTTAAAGTTTAAACA‑‑‑‑‑‑‑‑‑‑‑‑‑TAAAGACCAGACCTAAAGACCctagctc                                                                                                                                                                                                                                                                                            >  1:235647/1‑294`  
                     `GTGTACGGACCTAAAGTTCCACCATA‑GGGGGTACCTAAAGCCCAGCCAATCACCTAAAGTCAACCTTCGGTTGACCTTGAGGGTTCCCTAAGGGTTGGGGATGACCCTT‑GGGTTTGTCTTTGGGTGTTACCTTGAGTGTCTCTCTGTG‑T‑C‑CATATCTGTAACAGT‑‑‑‑‑‑‑‑‑‑‑‑‑CTCCTAAAGTATCCTCCTAAAGTCACCTCCTAACGTCCATCCTAAAGCCAACACCTAAAGCCTACACCTAAAGACCCATCAAGTCAACGCCTATCTTAAAGTTTAAACA‑‑‑‑‑‑‑‑‑‑‑‑‑TAAAGACCAGACCTAAAAACCACACCT                                                                                                                                                                                                                                                                                             >  1:80570/1‑301`  
                     `GTGTACGGACCTAAAGTTACCCCATA‑GGGGGTACCTAAAGCCCAGCCAATAACCTAAAGTCAACCTTCGGTTGACCTTGAGGGTTCCCTAAGGGTTGGGGATGACCCTT‑GGGTTTGTCTTTGGGTGTTACCTTGAGTGTCTCTCTGTG‑T‑C‑CC‑ATCTGTTACAGT‑‑‑‑‑‑‑‑‑‑‑‑‑CTCCTAAAGTATCCTCCTAAAGTCACCTCCTAACGTCCATCCTAAAGCAAACACCTAAAGCCTACACCTAAAGACCCATCAAGTCAACGCCTATCTTAAAGTTTAAACA‑‑‑‑‑‑‑‑‑‑‑‑‑TAAAGACCAGACCTAAAGACCAGACATA                                                                                                                                                                                                                                                                                            >  1:6767/1‑301`  
                       `GTACGGACCTAAAGTTTCCCCATAGGGGGGTACCTAAAGCCCAGCCAATCACCTAAAGTCAACCTTCGGTTGACCTTGAGGGTTCCCTAAGGGTTGGGGATGACCCTT‑GGGTTTGTCTTTGGGTGTTACCTTGAGTGTCTCTCTGTG‑TCC‑CCTATCTGTTACAGT‑‑‑‑‑‑‑‑‑‑‑‑‑CTCCTAAAGTATCCTCCTAAAGTCACCTACTAACGTCCATCCTAAAGCCAACACCTAAAGCCTACACCTAAAGACCCATCAAGTCAACGCCTATCTTAAAGTTTAAACA‑‑‑‑‑‑‑‑‑‑‑‑‑TAAAGACCAGACCTAAAGACCAGACCT                                                                                                                                                                                                                                                                                             >  1:31971/1‑301`  
                       `GTACGGACCTAAAGTTCCCCCATA‑GGGGGTACCTAAAGCCCAGCCAATCACCTAAAGTCAACCTTCGGTTGACCTTGAGGGTTCCCTAAGGGTTGGGGATGACCCTT‑GGGTTTGTCTTTGGGTGTTACCTTGAGTGTCTCTCTGTG‑TCC‑CCTATCTGTTACAGT‑‑‑‑‑‑‑‑‑‑‑‑‑CTCCTAAAGTATCCTCCTAAAGTCACCTCCTAACGTCCATCCTAAAGCCAACACCTAAAGCCTACACCTAAAGACCCATCAAGTCAACGCCTATCTTAAAGTTTAAACA‑‑‑‑‑‑‑‑‑‑‑‑‑TAAAGACCAGCACTAAAGACCAGACCTA                                                                                                                                                                                                                                                                                            >  1:213677/1‑301`  
                       `GTACGGACCTAAAGTTCCCCCATA‑GGGGGTACCTAAAGCCCAGCCAATCACCTAAAGTCAACCTTCGGTTGACCTTGAGGGTTCCCTAAGGGTTGGGGATGACCCTT‑GGGTTTGTCTTTGGGTGTTACCTTGAGTGTCTCTCTGTG‑T‑C‑CCTATCTGTTACAGT‑‑‑‑‑‑‑‑‑‑‑‑‑CTCCTAAAGTATCCTCCTAAAGTCACCTCCTAACGTCCATCCTAAAGCCAACACCTAAAGCCTACACCTAAAGACCCATCAAGTCAACGCCTATCTTAAAGTTTAAACA‑‑‑‑‑‑‑‑‑‑‑‑‑TAAAGACCAGACCTAAAGACtgtctcttt                                                                                                                                                                                                                                                                                           >  1:17753/1‑292`  
                       `GTACGGACCTAAAGTTCCCCCATA‑GGGGGTACCTAAAGCCCAGCCAATCACCTAAAGTCAACCTTCGGTTGACCTTGAGGGTTCCCTAAGGGTTGGGGATGACCCTT‑GGGTTTGTCTTTGGGTGTTACCTTGAGTGTCTCTCTGTG‑T‑C‑CCTATCTGTAACAGT‑‑‑‑‑‑‑‑‑‑‑‑‑CTCCTAAAGTATCCTCCTAAAGTCACCTCCTAACGTCCATCCTAAAGCCAACACCTAAAGCCTACACCTAAAGACCCATCAAGTCAACGCCTATCTTAAAGTTTAAACA‑‑‑‑‑‑‑‑‑‑‑‑‑TAAAGACCAGACCTAAAGctgtctcttat                                                                                                                                                                                                                                                                                           >  1:4114/1‑290`  
                       `GTACGGACCTAAAGTTCCCCCATA‑GGGGGTACCTAAAGCCCAGCCAATCACCTAAAGTCAACCTTCGGTTGACCTTGAGGGTTCCCTAAGGGTTGGGGATGACCCTT‑GGGTTTGTCTTTGGGTGTTACCTTGAGTGTCTCTCTGTG‑T‑C‑CCTATCTGTAACAGT‑‑‑‑‑‑‑‑‑‑‑‑‑CTCCTAAAGTATCCTCCTAAAGTCACCTCCTAACGTCCATCCTAAAGCCAACACCTAAAGCCTACACCTAAAGACCCATCAAGTCAACGCCTATCTTAAAGTTTAAACA‑‑‑‑‑‑‑‑‑‑‑‑‑TAAAGACCAGACCTAAAGACCAGACCTAA                                                                                                                                                                                                                                                                                           >  1:278762/1‑301`  
                       `GTACGGACCTAAAGTTCCCCCATA‑GGGGGTACCTAAAGCCCAGCCAATCACCTAAAGTCAACCTTCGGTTGACCTTGAGGGTTCCCTAAGGGTTGGGGATGACCCTT‑GGGTTTGTCTTTGGGTGTTACCTTGAGTGTCTCTCTGTG‑T‑C‑CCTATCTGTAACAGT‑‑‑‑‑‑‑‑‑‑‑‑‑CTCCTAAAGTATCCTCCTAAAGTCACCTCCTAACGTCCATCCTAAAGCCAACACCTAAAGCCTACACCTAAAGACCCATCAAGTCAACGCCTATCTTAAAGTTTAAACA‑‑‑‑‑‑‑‑‑‑‑‑‑TAAAGACCAGACCTAAAGACCAGACCTAA                                                                                                                                                                                                                                                                                           >  1:59636/1‑301`  
                       `GTACGGACCTAAAGTTCCCCCATA‑GGGGGTACCTAAAGCCCAGCCAATCACCTAAAGTCAACCTTCGGTTGACCTTGAGGGTTCCCTAAGGGTTGGGGATGACCCTT‑GGGTTTGTCTTTGGGTGTTACCTTGAGTGTCTCTCTGTG‑T‑C‑CCTATCTGTAACAGT‑‑‑‑‑‑‑‑‑‑‑‑‑CTCCTAAAGTATCCTCCTAAAGTAACCTCCTAACGTCCATCCTAAAGCCAACACCTAAAGCCTACACCTAAAGACCCATCAAGTCAACGCCTATCTTAACGTTTAAACA‑‑‑‑‑‑‑‑‑‑‑‑‑TAAAGACCctgtctcttatacacatctcc                                                                                                                                                                                                                                                                                           >  1:139902/1‑280`  
                       `GTACGGACCTAAAGTTCCCCCATA‑GGGGGTACCTAAAGCCCAGCCAATCACCTAAAGTCAACCTTCGGTTGACCTTGAGGGTTCCCTAAGGGTTGGGGATGACCCTT‑GGGTTTGTCTTTGGGTGTTACCTTGAGTGTCTCTCTGTG‑T‑C‑CCTATCTG‑TACAGT‑‑‑‑‑‑‑‑‑‑‑‑‑CTCCTAAAGTATCCTCCTAAAGTCACCTCCTAACGTCCATCCTAAAGCCAACACCTAAAGCCTACACCTAAAGACCCATCAAGTAAACGCCTATCTTAAAGTTTCAACA‑‑‑‑‑‑‑‑‑‑‑‑‑TAAAGACCAGACCTAAAGACCACACCTAAA                                                                                                                                                                                                                                                                                          >  1:154047/1‑301`  
                       `GTACGGACCTAAAGTTCCCCCATA‑GGGGGTACCTAAAGCCCAGCCAATCACCTAAAGTCAACCTTCGGTTGACCTTGAGGGTTCCCTAAGGGTTGGGGATGACCCTT‑GGGTTTGTCTTTGGGTGTTACCTTGAGTGTCTCTCTGTG‑T‑C‑CC‑ATCTGTTACAGT‑‑‑‑‑‑‑‑‑‑‑‑‑CTCCTAAAGTATCCTCCTAAAGTCACCTCCTAACGTCCATCCTAAAGCCAACACCTAAAGCCTACACCTAAAGACCCATCAAGTCAACGCCTATCTTAAAGTTTAAACA‑‑‑‑‑‑‑‑‑‑‑‑‑TAAAGACCAGACCTAAAGACCAGACCTAAA                                                                                                                                                                                                                                                                                          >  1:126497/1‑301`  
                       `GTACGGACCTAAAGTTCCCCCATA‑GGGGGTACCTAAAGCCCAGCCAATCACCTAAAGTCAACCTTCGGTTGACCTTGAGGGTTCCCTAAGGGTTGGGGATGACCCTT‑GGGTTTGTCTTTGGGTGTTACCTTGAGTGTCTCTCTGTG‑T‑C‑CC‑ATCTGTTACAGT‑‑‑‑‑‑‑‑‑‑‑‑‑CTCCTAAAGTATCCACCTAAAGTCACCACCTAACGTCCATCCTAAAGACAACACCTAAAGCACACACCTAAAGCCACATCAAGTCAAAGCCCATCTTAAAGTTTAAACA‑‑‑‑‑‑‑‑‑‑‑‑‑TAAAGACAAGACCGAAAGctgtatcttata                                                                                                                                                                                                                                                                                          >  1:326798/1‑289`  
                       `GTACGGACCTAAAGTTCCCCCATA‑GGGGGTACCTAAAGCCCAGCCAATCACCTAAAGTCAACCTTCGGTTGACCTTGAGGGTTCCCTAAGGGTTGGGGATGACCCTT‑GGGTTTGTCTTTGGGTGTTACCTTGAGTGTATCTCTGTG‑TCC‑CCTATCTGTTACAGT‑‑‑‑‑‑‑‑‑‑‑‑‑CTCCTAAAGTTTCCTCCTAAAGTCACCTCCTAACGTCCATCCTAAAGCCAACACCTAAAGCCTACACCTAAAGACCCATCAAGTCAACGCCTATCTTAAAGTTTAAACA‑‑‑‑‑‑‑‑‑‑‑‑‑TAAAGACCAGACCTAAAGACCAGACCTA                                                                                                                                                                                                                                                                                            >  1:341678/1‑301`  
                       `GTACGGACCTAAAGTTCCCCCATA‑GGGGGTACCTAAAGCCCAGCCAATCACCTAAAGTCAACCTTCGGTTGACCTTGAGGGTTCCATAAGGGTTGGGGATGACCCTT‑GGGTTTGTCTTTGGGTGTTACCTTGAGTGTCTCTCTGTG‑T‑C‑CCTATCTGTAACAGT‑‑‑‑‑‑‑‑‑‑‑‑‑CTCCTAAAGTATCCTCCTAAAGTCACCTCCTAACGTCAATCCTAAAGCCAACACCTAAAGCCTACACCTAAAGACCCATCAAGTCAACGCCTATCTTAAAGTTTAAACA‑‑‑‑‑‑‑‑‑‑‑‑‑TAAAGACCAGACCTAAAGACCAGACCctg                                                                                                                                                                                                                                                                                           >  1:261116/1‑298`  
                       `GTACGGACCTAAAGTTCCCCCATA‑GGGGGTACCTAAAGCCCAGCAAATCACCTAAAGTCAACCTTCGGTTGACCTTGAGGGTTCCCTAAGGGTTGGGGATGACCCTT‑GGGTTTGTCTTTGGGTGTTACCTTGAGTGTCTCTCTGTG‑T‑C‑CCTATCTGTAACAGT‑‑‑‑‑‑‑‑‑‑‑‑‑CTCCTAAAGTATCCTCCTAAAGTCACCTCCTAACGTCCATCCTAAAGCCAACACCTAAAGCCTACACCTAAAGACCCATCAAGTCAACGCCTATCTTAAAGTTTAAACA‑‑‑‑‑‑‑‑‑‑‑‑‑TAAAGACCAGACCTAAAGACCAGACCTAA                                                                                                                                                                                                                                                                                           >  1:69936/1‑301`  
                       `GTACGGACCTAAAGTTCCCACATA‑GGGGGTACCTAAAGCCCAGCCAATCACCTAAAGTCAACCTTCGGTTGACCTTGAGGGTTCCCTAAGGGTTGGGGATGACACTT‑GGGTTTGTCTTTGGGTGTTACCTTGAGTGTCTCTCTGTG‑T‑C‑CCTATCTG‑TACAGT‑‑‑‑‑‑‑‑‑‑‑‑‑CTCCTAAAGTATCCTCCTAAAGTCACCTCCTAACGTCCATCCTAAAGCCAACACCTAAAGCCTACACCTAAAGACCCATCAAGTCAACGCCTATCTTAAAGTTTAAACA‑‑‑‑‑‑‑‑‑‑‑‑‑TAAAGACCAGACCTAAAGACCAGACCTAAA                                                                                                                                                                                                                                                                                          >  1:121757/1‑301`  
                        `TACGGACCTAAAGTTCCCCCATA‑GGGGGTACCTAAAGCCCAGCCAATCACCTAAAGTCAACCTTCGGTTGTCCTTGAGGGTTCCCTAAGGGTTGGGGATGACCCTT‑GTGTTTGTCTTTGGGTGTTACCTTGAGTGTCTCTCTGTG‑T‑C‑CC‑ATCTGTTACAGT‑‑‑‑‑‑‑‑‑‑‑‑‑CTCCTAAAGTATCCTCCTAAAGTCACCTCCTAACGTCCATCCTAAAGCCAACACCTAAAGCCTACACCTAAAGACCCATCAAGTCAACGCCTATCTTAAAGTTTAAACA‑‑‑‑‑‑‑‑‑‑‑‑‑TAAAGACCAGACCTAAAGACCAGACCTAAAG                                                                                                                                                                                                                                                                                         <  1:283765/301‑1`  
                         `ACGGACCTAAAGTTCCCCCATA‑GGGGGTACCTAAAGCCCAGCCAATCACCTAAAGTCAACCTTCGGTTTACCTTGAGGGTTCCCTAAGGGTTGGGGATGACCCTT‑GGGTTTGTCTTTGGGTGTTACCTTGAGTGTCTCTCTGTG‑T‑C‑CCTATCTGTAACAGT‑‑‑‑‑‑‑‑‑‑‑‑‑CTCCTAAAGTATCCTCCTAAAGTCACCTCCTAACGTCCATCCTAAAGCCAACACCTAAAGCCTACACCTAAAGACCCATCAAGTCAACGCCTATCTTAAAGTTTAAACA‑‑‑‑‑‑‑‑‑‑‑‑‑TAAAGACCAGACCTAAAGACCAGACCTAAAG                                                                                                                                                                                                                                                                                         <  1:289630/301‑1`  
                         `ACGGACCTAAAGTTCCCCCAGA‑GGGGGTACCTAAAGCCCAGCCAATCACCTAAAGTCAACCTTCGGTTGACCTTGAGGGTTCCCTAAGGGTTGGGGATGACCCTT‑GGGTTTGTCTTTGGGTGTTACCTTGAGTGTCTCTCTGTG‑T‑C‑CCTATCTGTAACAGT‑‑‑‑‑‑‑‑‑‑‑‑‑CTCCTAAAGTATCCTCCTAAAGTCACCTCCTAACGTCCATCCTAAAGCCAACACCTAAAGCCTACACCTAAAGACCCATCAAGTCAACGCCTATCTTAAAGTTTAAACA‑‑‑‑‑‑‑‑‑‑‑‑‑TAAAGACCAGACCTAAAGACCAGACCTAAAG                                                                                                                                                                                                                                                                                         <  1:133256/301‑1`  
                         `ACGGACCTAAAGTTCCCACATA‑GGGGGTACCTAAAGCCCAGCCAATCACCTAAAGTCAACCTTCGCTTGACCTTGAGGGTTCCCTAAGGGTTGGGGATGACCCTT‑GGGTTTGTCGTTGGGTGTTACCTTGAGTGTCTCTCTGGT‑T‑C‑CCTATCTGTAACAGT‑‑‑‑‑‑‑‑‑‑‑‑‑CTCCTAAAGTATCCTCCTAAAGTCACCTCCTAACGTCCATCCTAAAGCCAACACCTAAAGACTACACCTAAAGACCCATCAAGTCAACACCTATCTTAAAGTTTAAACA‑‑‑‑‑‑‑‑‑‑‑‑‑TAAAGACCAGACCTAAAGACCAGAACTAAAG                                                                                                                                                                                                                                                                                         <  1:182792/301‑1`  
                           `ggtgtataagagacagCATA‑GGGTGTACCTAAAGCCCAGCCAATCACCTAAAGTCAACCTTCGGTTGACCTTGAGGGTTCCCTAAGGGTTGGGGATGACCCTT‑GGGTTTGTCTTTGGGTGTTACCTTGGTTGTCTCTCTGTG‑T‑C‑CCTATCTGTAACAGT‑‑‑‑‑‑‑‑‑‑‑‑‑CTCCTAAAGTATCCTCCTAAAGTCACCTCCTAACGTCCATCCTAAAGCCAACACCTAAAGCCTACACCTAAAGACCCATCAAGTCAACGCCTATCTTAAAGTTTAAACA‑‑‑‑‑‑‑‑‑‑‑‑‑TAAAGACCAGACCTAAAGACCAGACCTAAAGAC                                                                                                                                                                                                                                                                                       <  1:317425/285‑1`  
                           `GGACCTAAAGTTCCCCTATA‑GGGGGTACCTAAAGCCCAGCCAATCACCTAAAGTCAACCTTCGGTTGACCTTGAGGGTTCCCTAAGGGTTGGGGATGACCCTT‑GGGTTTGTCTTTGGGTGTTACCTTGAGTGTCTCTCTGTG‑T‑C‑CCTATCTGTAACAGT‑‑‑‑‑‑‑‑‑‑‑‑‑CTCCTAAAGTATCCTCCTAAAGTCACCTCCTAACGTCCATCCTAAAGCCAACACCTAAAGCCTACACCTAAAGACCCATCAAGTCAACGCCTATCTTAAAGTTTAAACA‑‑‑‑‑‑‑‑‑‑‑‑‑TAAAGACCAGACCTAAAGACCAGACCTAAAGAC                                                                                                                                                                                                                                                                                       <  1:4728/301‑1`  
                           `GGACCTAAAGTTCCCCCATA‑GGGGGTACCTAAAGCCCAGCCAATCACCTAAAGTCAACCTTCGGTTGACCTTGAGGGTTCCCTAAGGGTCGGGGATGACCCTT‑GGGTTTGTCTTTGGGTGTCACCTTGAGTGTCTCTCTGTG‑T‑C‑CCTATCTGTAACAGT‑‑‑‑‑‑‑‑‑‑‑‑‑CTCCTAAAGTATCCTCCTAAAGTCACCTCCTAACGTCCATCCTAAAGCCAACACCTAAAGCCTACACCTAAAGACCCATCAAGTCAACGCCTATCTTAAAGTTTAAACA‑‑‑‑‑‑‑‑‑‑‑‑‑TAAAGACCAGACCTAAAGACCAGACCTAAAGAC                                                                                                                                                                                                                                                                                       <  1:93554/301‑1`  
                           `GGACCTAAAGTTCCCCCATA‑GGGGGTACCTAAAGCCCAGCCAATCACCTAAAGTCAAACTTCGGTTGACCTTGAGGGTTCCCTAAGGGTTGGGGATGACCCTT‑GGGTTTGTCTTTGGGTGTTACCTTGAGTGTCTCTCTGTG‑T‑C‑CCTATCTGTAACAGT‑‑‑‑‑‑‑‑‑‑‑‑‑CTCCTAAAGTATCCTCCTAAAGTCACCTCCTAACGTCCATCCTAAAGCAAACACCTAAAGCCTACACCTAAAGACCCATCAAGTCAACGCCTATCTTAAAGTTTAAACA‑‑‑‑‑‑‑‑‑‑‑‑‑TAAAGACCAGACCTAAAGACCAGACCTAAAGAC                                                                                                                                                                                                                                                                                       <  1:37811/301‑1`  
                           `GGACCTAAAGTTCCCCAATA‑GGGGGTACCTAAAGCCCAGCCAATCACCTAAAGTCAACCTTCGGTTGACCTTGAGGGTTCCCTAAGGGTTGGGGATGACCCTT‑GGGTTTGTCTTTGGGTGTTACCTTGAGTGTCTCTCTGTG‑T‑C‑CCTATCTGTAACAGT‑‑‑‑‑‑‑‑‑‑‑‑‑CTCCTAAAGTATCCTCCTAAAGTCACCTCCTAACGTCCATCCTAAAGCCAACACCTAAAGCCTACACCTAAAGACCCATCAAGTCAACGCCTATCTTAAAGTTTAAACA‑‑‑‑‑‑‑‑‑‑‑‑‑TAAAGACCAGACCTAAAGACCAGACCTAAAGAC                                                                                                                                                                                                                                                                                       <  1:168572/301‑1`  
                           `GGACCTAAAGTCCCCCCAGA‑GGGGTGACCTAAAGCCCAGCCAATCACCTAAAGTCAACCTTCGGTTGACCTTGAGGGTTCCCTAAGGGTTGGGGATGACCCTT‑GGGTTTGTCTTTGGGTGTTACCTTGAGTGTCTCTCTGTG‑T‑C‑CCTATCTGTAACAGT‑‑‑‑‑‑‑‑‑‑‑‑‑ATCCTAAAGTATCCTCCTAAAGTCACCTCCTAACGTCCATCCTAAAGCCAACACCTAAAGCCTACACCTAAAGACCCATCAAGTCAACGCCTATCTTAAAGTTTAAACA‑‑‑‑‑‑‑‑‑‑‑‑‑TAAAGACCAGACCTAAAGACCAGACCTAAAGAC                                                                                                                                                                                                                                                                                       <  1:41672/301‑1`  
                            `GACCTAAAGTTCCCCCATA‑GGGTGTACCTAAAGCCTAGCCAATCACCTAAAGTCAACCTTCGGTTGACCTTGAGGGTTCCCTAAGGGTTGGGGATGACCCTT‑GGGTTTGTCTTTGGGTGTTACCTTGAGTGTCTCTCTGTG‑T‑C‑CCTATCTGTAACAGT‑‑‑‑‑‑‑‑‑‑‑‑‑CTCCTAAAGTATCCTCCTAAAGTCACCTCCTAACGTCCATCCTAAAGCCAACACCTAAAGCCTACACCTAAAGACCCATCAAGTCAACGCCTATCTTAAAGTTTAAACA‑‑‑‑‑‑‑‑‑‑‑‑‑TAAAGACCAGACCTAAAGACCAGACCTAAAGACC                                                                                                                                                                                                                                                                                      >  1:201093/1‑301`  
                            `GACCTAAAGTTCCCCCATA‑GGGGGTACCTAAAGCCCAGCCAATCACCTAAAGTCAACCTTCGGTTGACCTTGAGGGTTCCCTAAGTGTTGGGGATGACCCTT‑GGGTTTGTCTTTGGGTGTTACCTTGAGTGTCTCTCTGTG‑T‑C‑CCTATCTGTAACAGT‑‑‑‑‑‑‑‑‑‑‑‑‑CTCCTAAAGTATCCTCCTAAAGTCACCTCCTAACGTCCATCCTAAAGCCAACACCTAAAGCCTACACCTAAAGACCCATCAAGTCAACGCCTATCTTAAAGTTTAAACA‑‑‑‑‑‑‑‑‑‑‑‑‑TAAAGACCAGACCTAAAGACCAGACCctgtctct                                                                                                                                                                                                                                                                                      >  1:16325/1‑293`  
                            `GACCTAAAGTTCCCCCATA‑GGGGGTACCTAAAGCCCAGCCAATCACCTAAAGTCAACCTTCGGTTGACCTTGAGGGTTCCCTAAGGGTTGGGGATGACCCTT‑GGTTTTGTCTTTGGGTGTTACCTTGAGTGTCTCTCTGTG‑T‑C‑CCTATCTGTAACATT‑‑‑‑‑‑‑‑‑‑‑‑‑CTCCTAAAGTATCCTCCTAAAGTCACCTACTAACGTCCATCCTAAAGCCAACACCTAAAGCCTACACCTAAAGCCCCATCAAGTCAACGCCTATCTTAAAGTTTAAACA‑‑‑‑‑‑‑‑‑‑‑‑‑TAAAGACCAGACCTAAAGACCAGACCTAAAGACC                                                                                                                                                                                                                                                                                      >  1:165173/1‑301`  
                            `GACCTAAAGTTCCCCCATA‑GGGGGTACCTAAAGCCCAGCCAATCACCTAAAGTCAACCTTCGGTTGACCTTGAGGGTTCCCTAAGGGTTGGGGATGACCCTT‑GGGTTTGTCTTTGGTTGTTACCTTGAGTGTCTCTCTGTC‑T‑C‑CCTATCTGTAACAGT‑‑‑‑‑‑‑‑‑‑‑‑‑CTCCTAAAGTATCCTCCTAAAGTCACCTCCTAACGTCCATCCTAAAGCCAACACCTAAAGCCTACACCTAAAGAACCCTCAAGTCAACGCCTATCTTAAAGTTTAAACA‑‑‑‑‑‑‑‑‑‑‑‑‑TAAAGctgtatattatacacatctcagatcccaa                                                                                                                                                                                                                                                                                      >  1:76326/1‑272`  
                            `GACCTAAAGTTCCCCCATA‑GGGGGTACCTAAAGCCCAGCCAATCACCTAAAGTCAACCTTCGGTTGACCTTGAGGGTTCCCTAAGGGTTGGGGATGACCCTT‑GGGTTTGTCTTTGGGTGTTACCTTGAGTGTCTCTCTGTG‑T‑C‑CCTATCTGTAACAGT‑‑‑‑‑‑‑‑‑‑‑‑‑CTCCTAAAGTATCCTCCTAAAGTCACCTCCTAACGTCCATCCTAAAGCCAACACCTAAAGCCTACACCTAAAGACCCATCAAGTCAACGCCTATCTTAAAGTTTAAACA‑‑‑‑‑‑‑‑‑‑‑‑‑TAAAGACCtgtctcttatacacatctccgagccc                                                                                                                                                                                                                                                                                      >  1:207909/1‑275`  
                            `GACCTAAAGTTCCCCCATA‑GGGGGTACCTAAAGCCCAGCCAATCACCTAAAGTCAACCTTCGGTTGACCTTGAGGGTTCCCTAAGGGTTGGGGATGACCCTT‑GGGTTTGTCTTTGGGTGTTACCTTGAGTGTCTCTCTGTG‑T‑C‑CCTATCTGTAACAGT‑‑‑‑‑‑‑‑‑‑‑‑‑CTCCTAAAGTATCCTCCTAAAGTCACCTCCTAACGTCCATCCTAAAGCCAACACCTAAAGCCTACACCTAAAGACCCATCAAGTCAACGCCTATCTTAAAGTTTAAACA‑‑‑‑‑‑‑‑‑‑‑‑‑TAAAGACCAGACCTAAAGACCtgtctcttataca                                                                                                                                                                                                                                                                                      >  1:113179/1‑288`  
                            `GACCTAAAGTTCCCCCATA‑GGGGGTACCTAAAGCCCAGCCAATCACCTAAAGTCAACCTTCGGTTGACCTTGAGGGTTCCCTAAGGGTTGGGGATGACCCTT‑GGGTTTGTCTTTGGGTGTTACCTTGAGTGTCTCTCTGTG‑T‑C‑CCTATCTGTAACAGT‑‑‑‑‑‑‑‑‑‑‑‑‑CTCCTAAAGTATCCTCCTAAAGTCACCTCCTAACGTCCATCCTAAAGCCAACACCTAAAGCCTACACCTAAAGACCCATCAAGTCAACGCCTATCTTAAAGTTTAAACA‑‑‑‑‑‑‑‑‑‑‑‑‑TAAAGACCAGACCTAAAGACCAGACCctgtctct                                                                                                                                                                                                                                                                                      >  1:199241/1‑293`  
                            `GACCTAAAGTTCCCCCATA‑GGGGGTACCTAAAGCCCAGCCAATCACCTAAAGTCAACCTTCGGTTGACCTTGAGGGTTCCCTAAGGGTTGGGGATGACACTT‑GGGTTTGTCTTTGGGTGTTACCTTGCGTGTCTCTCTGTG‑T‑C‑CCTATCTGTAACGGT‑‑‑‑‑‑‑‑‑‑‑‑‑CTCCTAAGGTATCCTCCTAAAGTCACCTCCTAAGGTCCATCCTAAAGCCAACACCTAATGCATACACCTAAAGACCCATCCATTCCACGCCTCTCTTTAAGTTTAAACA‑‑‑‑‑‑‑‑‑‑‑‑‑TAAAGAACACACCTAAAGACCAGACCTAAcacac                                                                                                                                                                                                                                                                                      >  1:26820/1‑296`  
                            `GACCTAAAGTTCCCCCATA‑GGGGGTACCTAAAGCCCAGCCAATCACCTAAAGTCAACCTTCGGTTGACATTGAGGGTTCCCTAAGGGTTTGTGATGACCCTT‑GGTTTTGTCTTTGGGTGTTACCTTGATTGTCTCTCTGTG‑T‑C‑CCTATCTGTAACAGT‑‑‑‑‑‑‑‑‑‑‑‑‑CTCCTAAAGTATCCTCCTAAAGTCACCTCCTCACGTCCATCCTAAAGCCAACACCTAAAGCCTACACCTAAAGACCCATCAAGTCAACGCCTATCTTAAAGTTTAAACA‑‑‑‑‑‑‑‑‑‑‑‑‑TAAAGACCAGACCTAAATACCAGACCTAAAGcca                                                                                                                                                                                                                                                                                      >  1:215043/1‑298`  
                            `GACCTAAAGATCCCCCATA‑GGGTGTACCTAAAGCCCAGCCAATCACCTAAAGTCAACCTTCGGTTGACCTTGAGGGTTCCCTAAGGGTTGGGGATGACCCTT‑GGGTTTGTCTTTGGGTGTTACCTTGAGTGTCTCTCTGTG‑T‑C‑CCTATCTGTAACAGT‑‑‑‑‑‑‑‑‑‑‑‑‑ATCCTAAAGTATCCTCCTAAAGTCACCTCCTACCGTCCATCCTAAAGCCACCACCTAAAGCCTACACCTAAAGACCAATCAAGTCAACGCCTATCTTAAAGTTTAAACA‑‑‑‑‑‑‑‑‑‑‑‑‑TAAAGACCAGACATAAAGACCAGACCTAAcgctg                                                                                                                                                                                                                                                                                      >  1:292249/1‑296`  
                             `ACCTAAAGTTCCCCCATA‑GGGGGTACCTAAAGCCCAGCCAATCACCTAAAGTCAACCTTCGGTTGACCTTGAGGGTTCCCTAAGGGTTGGGGATGACCCTT‑GGGTTTGTCTTTGGGTGTTACCTTGAGTGTCTCTCTGTG‑TCC‑CCTATCTGTTACAGT‑‑‑‑‑‑‑‑‑‑‑‑‑CTCCTAAAGTATCCTCCTAAAGTCACCTCCTAACGTCCATCCTAAAGCCAACACCTAAAGCCTACACCTAAAGACCCATCAAGTCAACGCCTATCTTAAAGTTTAAACA‑‑‑‑‑‑‑‑‑‑‑‑‑TAAAGACCAGACCTAAAGACCctgtctcttatac                                                                                                                                                                                                                                                                                      >  1:55041/1‑288`  
                              `CCTAAAGTTCCCCCATA‑GGGGGTACCTAAAGCCCAGCCAATCACCTAAAGTCAACCTTCGGTTGACCTTGAGGGTTCCCTAAGGGTTGGGGATGACCCTT‑GGGTTTGTCTTTGGGTGTTACCTTGAGTGTCTCTCTGTG‑T‑C‑CCTATCTGTTACAGTCTCCTAAAGTATCCTCCTAAAGTATCCTCCTAAAGTCACCTCCTAACGTCCATCCTAAAGCCAACACCTAAAGCCTACACCTAAAGACCCATCAAGTCAACGCCTATCTTAAAGTTTAAACA‑‑‑‑‑‑‑‑‑‑‑‑‑TAAAGACCAGACCctgtctctta                                                                                                                                                                                                                                                                                                 >  1:305071/1‑291`  
                                       `CCCCCATA‑GGGGGTACCTAAAGCCCAGCCAATCACCTAAAGTCAACCTTCGTTTGACCTTGAGGGTTCACTAAGGGTTGGGGATGACCCTT‑GGGTTTGTCTTTGGGTGTTACCTTGAGTGTCTCTCTGTG‑T‑C‑CCTATCTG‑TACAGT‑‑‑‑‑‑‑‑‑‑‑‑‑CTCCTAAAGTATCCTCCTAAAGTCACCTCCTAACGTCCATCCTAAAGCCAACACCTAAAGCCTACAACTAAAGACCCATCAAGTCAACGCCTATCTT‑TAGTTTAAACA‑‑‑‑‑‑‑‑‑‑‑‑‑TAAAGACCAGACCTAAAGACCtgtctcttatacacatctccgagccc                                                                                                                                                                                                                                                                         >  1:194005/1‑275`  
                                                      `ACCTAAAGCCCAGCCAATCACCTAAAGTCCACCTTCGGTTGACCTTGAGGGTTCCCTAAGGGTTGGGGATGACCCTT‑GGGTTTGTCTTTGGGTGTTACCTTGAGTGTCTCTCTGTG‑TCC‑CCTATCTGTTACAGT‑‑‑‑‑‑‑‑‑‑‑‑‑CTCCTAAAGTATCCTCCTAAAGTCACCTCCTAACGTCCATCCTAAAGCCAACACCTAAAGCCTACACCTAAAGACCCATCAAGTCAACGCCTATCTTAAAGTTTAAACA‑‑‑‑‑‑‑‑‑‑‑‑‑TAAAGACCAGACCTAAAGACCAGACCTAAAGACCAGACCTAAAGACACTACATAAAGA                                                                                                                                                                                                                                                              <  1:306159/301‑1`  
                                                      `ACCTAAAGCCCAGCCAATCACCTAAAGTCAACCTTCGGTTGACCTTGAGGGTTCCCTAAGGGTTGGGGATGACCCTT‑GGGTTTGTCTTTGGGTGTTACCTTGAGTGTCTCTCTGTG‑T‑C‑CCTATCTGTAACAGT‑‑‑‑‑‑‑‑‑‑‑‑‑CTCCTAAAGTATCCTCCTAAAGTCACCTCCTAACGTCCATCCTAAAGCCAACACCTAAAGCCTACACCTAAAGACCCATCAAGTCAACGCCTATCTTAAAGTTTAAACA‑‑‑‑‑‑‑‑‑‑‑‑‑TAAAGACCAGACCTAAAGACCAGACCTAAAGACCtgtctcttatacacatctccgagcc                                                                                                                                                                                                                                                             >  1:211565/1‑276`  
                                                                                                                    `TTGGGGATGAACATT‑GGGTTGTGCTTTTGGTGTTACCTTGAGTGTCTCTCTGTG‑TCC‑CCTATCTGTTACAGT‑‑‑‑‑‑‑‑‑‑‑‑‑CTCCTAAAGTATCCTCCTAAAGTAACCTCCTAACGTCCATCCTAAAGCCAACACCTAAAGCATACACCTAAAGACCCATCAAGTCAACGCCTATCTTAAAGTTTAAACA‑‑‑‑‑‑‑‑‑‑‑‑‑TAAAGACCAGACCTAAAGACCAGACCTAAAGACCAGACCTAAAGACACTACATAAAGACCAGACCTAAAGACGCCTTGTTGTTAGCCATAAAGTGATAACCTTTAATCATTGTCTTTATT                                                                                                                                                                                                <  1:116029/301‑1`  
                                                                                                                                                  `GTGTTACCTTGAGTGTCTCTCTGTG‑T‑C‑CCTATCTGTAACAGT‑‑‑‑‑‑‑‑‑‑‑‑‑CTCCTAAAGTATCCTCCTAAAGTCACCTCCTAACGTCCATCCTAAAGCCAACACCTAAAGCCTACACCTAAAGACCCATCAAGTCAACGCCTATCTTAAAGTTTAAACA‑‑‑‑‑‑‑‑‑‑‑‑‑TAAAGACCAGACCTAAAGACCAGACCTAAAGACCAGACCTAAAGACACTACATAAAGACCAGACCTAAAGACGCCTTGTTGTTAGACATAAAGTGATAACCTTTAATCATTGTCTTTATTAATACAACTCACTATAAGGAGAGACAACTT                                                                                                                                                                  >  1:218403/1‑301`  
                                                                                                                                                                                   `ATCTGTAACAGT‑‑‑‑‑‑‑‑‑‑‑‑‑CTCCTAAAGTATCCTCCTAAAGTCACCTCCTAACGTCCATCCTAAAGCCAACACCTAAAGCCTACACCTAAAGACCCATCAAGTCAACGCCTATCTTAAAGTTTAAACA‑‑‑‑‑‑‑‑‑‑‑‑‑TAAAGACCAGACCTAAAGACCAGACCTAAAGACACTACATAAAGACACTACATAAAGACCAGACCTAAAGACGCCTTGTTGTTAGCCATAAAGTGATAACCTTTAATCCTTATCTTTATTAATACAACTCACTATAAGGAGAGACACCTTAAAGAGCCTTAAAAGATTAATTTAAAATTT                                                                                                                                    >  1:73619/1‑301`  
                                                                                                                                                                                                                 `AAAGTATCCTCCTAAAGTCACCTCCTAACGTCCATCCTAAAGCCAACACCTAAAGCCTACACCTAAAGACCCATCAAGTCAACGCCTATCTTAAAGTTTAAACA‑‑‑‑‑‑‑‑‑‑‑‑‑TAAAGACCAGACCTAAAGACCAGACCTAAAGACCAGACCTAAAGACACTACATAAAGACCAGACCTACAGACGCCTTGTTGTTAGCCATAAAGTGATAACCTTTAATCATTGTCTTTATTAATACAACTCACTATAAGTAGAGACAACTTAAAGAGACTTAAAAGATTAATTTAAAATTTATCAAAACGAGTATTta                                                                                                                   >  1:334015/1‑299`  
                                                                                                                                                                                                                                `AGTCACCTCCTAACGTCCATCCTAAAGCCAACACCTAAAGCCTACACCTAAAGACCCATCAAGTCAACGCCTATCTTAAAGTTTAAACA‑‑‑‑‑‑‑‑‑‑‑‑‑TAAAGACCAGACCTAAAGACCAGACCTAAAGACCAGACCTAAAGACACTACATAAAGACCAGACCTAAAGACGCCTTGTTGTTAACCATAAAGTGATAACCTTTAATCATTGTCTTTATTAATACAACTCACTATAAGGAGAGACAACTTAAAGAGACTTAAAAGATTAATTTAAAATTTATCAAAAAGAGTATTGACTTAAAGTCTAACCT                                                                                                    >  1:228871/1‑301`  
                                                                                                                                                                                                                                                `cagTCCTAAAGCCAACACCTAAAGCCTACTCCTAAAGACCCATCAAGTCAACGCCTATCTTAAAGTTTAAACA‑‑‑‑‑‑‑‑‑‑‑‑‑TAAAGACCAGACCTAAAGACCAGACCTAAAGACCAGACCTAAAGACACTACATAAAGACCAGACCTAAAGACGCCTTGTTGTTAGCCATAAAGTGATAACCTTTAATCATTGTCTTTATTAATACAACTCACTATAAGGAGAGACAACTTAAAGAGACTTAAAAGATTAATTTAAAATTTATCAAAAAGAGTATTGACTTAAAGTCTAACCTATAGGATACTTACAGC                                                                                    <  1:152609/298‑1`  
                                                                                                                                                                                                                                                          `atacagACCTAAATCCTACACCTAAAGACCCATCAAGTCAACGCCGATCTTAAAGTTTAAACATAAAGTCCAGACCTAAAGACCAGACCTAAAGACCAGACCTAAAGACCAGACCTAAAGACACTACATAAAGACCAGACCTAAAGACGCCTTTTTGTTAGCCATAAATTGATAACCTTTAATCATTGTCTTTATTAATACAACTCACTATAAGGAGAGACAACTTAAAGAGACTTAAAAGATTAATTTAAAATTTATCAAAAAGAGTATTGACTTAAAGTCTAACCTATAGGATACTTAC                                                                                       <  1:81680/295‑1`  
                                                                                                                                                                                                                                                                 `atataagagacagCCTAAAGACCCATCAAGTCAACGCCGATCTTAAAGTTTAAACA‑‑‑‑‑‑‑‑‑‑‑‑‑TAAAGACCAGACCTAAATACCAGACCTAAAGACCAGACCTAAAGACACTACATAAAGACCAGACCTAAAGACGCCTTGTTGTTAGCCATAAAGTGATAACCTTTAATCATTGTCTTTATTAATACAACTCACTATAAGGAGAGACAACTTAAAGAGACTTAAAAGATTAATTTAAAATTTATCAAAAAGAGTATTGACTTAAAGTCTAACCTATAGGATACTTACAGCCATCGAGAGGGACACGG                                                                   <  1:281052/288‑1`  
                                                                                                                                                                                                                                                                              `CCTCAAGACCCATCAAGTCAACGCCTATCTTAAAGTTTAAACA‑‑‑‑‑‑‑‑‑‑‑‑‑TAAAGACCAGACCTAAAGACCAGACCTAAAGACCAGACCTAAAGACACTACATAAAGACCAGACCTAAAGACGCCTTGTTGTTAGCCATAAAGTGATAACCTTTAATCATTGTCTTTATTAATACAACTCACTATAAGGAGAGAAAACTTAAAGAGACTTAAAAGATTAATTTAAAATTTATCAAAAAGAGTATTGACTTAAAGTCTAACCTATAGGATACTTACAGCCATCGAGAGGGctgtctcttatacacatct                                                      >  1:307346/1‑282`  
                                                                                                                                                                                                                                                                                                                                               `GACCTAAAGACCAGACCTAAAGACCAGACCTAAAGACACTACATAAAGACCAGACCTAAAGACGCCTTGTTGTTAGCCATAAAGTGATAACCTTTAATCATTGTCTTTATTAATACAACTCACTATAAGGAGAGACAGCTTAAAGAGACTTAAAAGATTAATTTAAAATTTATCAAAAAGAGTATTGACTTAAAGTCTAACCTATAGGATACTTACAGCTATCGCGAGGGACACGTCGAATAGCCATCCCAATCGACACTGGTGTCAACCGGTTAAGTAGACAGCCTGATAActgtctctt  >  1:143996/1‑292`  

                                                                                                                                                                                                                                                                                                                         `||||||||||||||||||||||||||`                                                                                                                                                                                                                                                                                                           
`‑‑‑‑‑‑‑‑‑‑‑‑‑‑TCTCACAGTGTACGGACCTAAAGTTCCCCCATA‑GGGGGTACCTAAAGCCCAGCCAATCACCTAAAGTCAACCTTCGGTTGACCTTGAGGGTTCCCTAAGGGTTGGGGATGACCCTT‑GGGTTTGTCTTTGGGTGTTACCTTGAGTGTCTCTCTGTG‑T‑C‑CCTATCTGTTACAGT‑‑‑‑‑‑‑‑‑‑‑‑‑CTCCTAAAGTATCCTCCTAAAGTCACCTCCTAACGTCCATCCTAAAGCCAACACCTAAAGCCTACACCTAAAGACCCATCAAGTCAACGCCTATCTTAAAGTTTAAACA‑‑‑‑‑‑‑‑‑‑‑‑‑‑‑‑‑‑‑‑‑‑‑‑‑‑‑‑‑‑‑‑‑‑‑‑‑‑‑‑‑‑‑‑‑‑‑‑‑‑‑‑‑‑‑‑‑‑‑‑‑‑‑‑‑‑‑‑‑‑‑‑‑‑‑‑‑‑‑‑‑‑‑‑‑‑‑‑‑‑‑‑‑‑‑‑‑‑‑‑‑‑‑‑‑‑‑‑‑‑‑‑‑‑‑‑‑‑‑‑‑‑‑‑‑‑‑‑‑‑‑‑‑‑‑‑‑‑‑‑‑‑‑‑‑‑‑‑‑‑‑‑‑‑‑‑‑‑‑‑‑‑‑‑‑‑‑‑‑‑‑‑‑‑‑‑‑‑‑‑‑‑‑‑‑‑‑‑‑‑‑‑‑‑‑‑‑‑‑‑‑‑‑‑‑‑‑‑‑‑‑‑‑‑‑‑‑‑‑‑‑‑‑‑‑‑‑‑‑‑‑‑‑‑‑‑‑‑‑‑‑‑‑‑‑‑‑‑‑‑‑‑‑‑‑‑‑‑‑‑‑‑‑‑‑‑‑‑‑‑‑‑‑‑‑‑‑‑‑‑‑‑‑‑‑‑‑‑‑‑‑‑‑‑‑‑‑‑‑‑‑‑‑‑‑‑‑‑‑‑‑‑‑‑‑‑‑‑‑‑‑‑‑  >  NC_001604.1/1‑281`  
`‑‑‑‑‑‑‑‑‑‑‑‑‑‑‑‑‑‑‑‑‑‑‑‑‑‑‑‑‑‑‑‑‑‑‑‑‑‑‑‑‑‑‑‑‑‑‑‑‑‑‑‑‑‑‑‑‑‑‑‑‑‑‑‑‑‑‑‑‑‑‑‑‑‑‑‑‑‑‑‑‑‑‑‑‑‑‑‑‑‑‑‑‑‑‑‑‑‑‑‑‑‑‑‑‑‑‑‑‑‑‑‑‑‑‑‑‑‑‑‑‑‑‑‑‑‑‑‑‑‑‑‑‑‑‑‑‑‑‑‑‑‑‑‑‑‑‑‑‑‑‑‑‑‑‑‑‑‑‑‑‑‑‑‑‑‑‑‑‑‑‑‑‑‑‑‑‑‑‑‑‑‑‑‑‑‑‑‑‑‑‑‑‑‑‑‑‑‑‑‑‑‑‑‑‑‑‑‑‑‑‑‑‑‑‑‑‑‑‑‑‑‑‑‑‑‑‑‑‑‑‑‑‑‑‑‑‑‑‑‑‑‑‑‑‑‑‑‑‑‑‑‑‑‑‑‑‑‑‑‑‑‑‑‑‑‑‑‑‑‑‑‑‑‑‑‑‑‑‑‑‑‑‑‑‑‑‑‑‑‑‑‑‑‑‑‑‑‑‑‑‑‑‑‑‑‑‑‑‑‑‑‑‑‑‑‑‑‑‑‑‑‑‑‑‑‑‑‑‑‑‑‑‑‑‑‑‑‑‑TAAAGACCAGACCTAAAGACCAGACCTAAAGACACTACATAAAGACCAGACCTAAAGACGCCTTGTTGTTAGCCATAAAGTGATAACCTTTAATCATTGTCTTTATTAATACAACTCACTATAAGGAGAGACAACTTAAAGAGACTTAAAAGATTAATTTAAAATTTATCAAAAAGAGTATTGACTTAAAGTCTAACCTATAGGATACTTACAGCCATCGAGAGGGACACGGCGAATAGCCATCCCAATCGACACCGGGGTCAACCGGATAAGTAGACAGCCTGATAAGTCGCACGA  >  NC_001604.1/282‑578`

  

Alignment Legend

Aligned base mismatch/match (shaded by quality score): `ATCG/ATCG` `< 3 ≤` `ATCG/ATCG` `< 11 ≤` `ATCG/ATCG` `< 23 ≤` `ATCG/ATCG` `< 35 ≤` `ATCG/ATCG` `< 38 ≤` `ATCG/ATCG`

Unaligned base: `atcg`    Masked matching base: `atcg`    Alignment gap: `‑`    Deleted base: `‑`

Reads not counted as support for junction

read\_name Not counted due to insufficient overlap past the breakpoint.

read\_name Not counted due to not crossing MOB target site duplication.
